# Physical Activity–Sedentary Behaviour Profiles and Co-Occurring Lifestyle Behaviours Among Adolescents: A Person-Oriented Analysis

**DOI:** 10.3390/nu18172927

**Published:** 2026-09-07

**Authors:** Mariola Wojciechowska, Arkadiusz Łukasz Żurawski, Elżbieta Cieśla, Anna Zmyślna, Dorota Kozieł

**Affiliations:** 1 Department of Social Problems and Social Work, Institute of Pedagogy, Faculty of Pedagogy and Psychology, Jan Kochanowski University in Kielce, Krakowska 11, 25-029 Kielce, Poland; mariola.wojciechowska@ujk.edu.pl; 2 Department of Physiotherapy, Institute of Health Sciences, Faculty of Health Sciences, Collegium Medicum, Jan Kochanowski University in Kielce, al. IX Wieków Kielc 19A, 25-516 Kielce, Poland; anna.zmyslna@ujk.edu.pl; 3 Department of Nutrition and Dietetics, Institute of Health Sciences, Faculty of Health Sciences, Collegium Medicum, Jan Kochanowski University in Kielce, al. IX Wieków Kielc 19A, 25-516 Kielce, Poland; elzbieta.ciesla@ujk.edu.pl; 4 Department of Nursing and Other Health Professions, School of Public Health, Centre of Postgraduate Medical Education, Marymoncka 99/103, 01-813 Warsaw, Poland; dorota.koziel@cmkp.edu.pl

**Keywords:** adolescents, physical activity, sedentary behaviour, cluster analysis, dietary behaviours, sleep, television/computer use

## Abstract

**Background/Objectives**: Physical activity and sedentary behaviour are important components of adolescent lifestyle, but favourable movement behaviour may not correspond to favourable behaviours across other health-related domains. This study aimed to identify empirical physical activity–sedentary behaviour profiles among adolescents and examine their co-occurring dietary, sleep, digital, and general health behaviours. **Methods**: This cross-sectional, school-based study used data from 3307 Polish adolescents aged 15–17 years. Following final processing of the International Physical Activity Questionnaire–Long Form, 1932 participants had complete and analytically eligible data for sedentary time, walking, moderate-intensity physical activity, and vigorous-intensity physical activity and were included in profile derivation. The four variables were Z-standardised before k-means clustering. Solutions from k = 2 to k = 6 were evaluated using the silhouette coefficient, Calinski–Harabasz index, Davies–Bouldin index, and Adjusted Rand Index (ARI). Co-occurring behaviours were compared using chi-square and Mann–Whitney U tests with effect-size estimates. **Results**: Cluster assignments were almost identical across repeated k-means runs initiated from different random starting centroids (ARI = 0.999). Because all runs were performed on the same set of participants, this result reflects low dependence on the initial centroid configuration rather than robustness to changes in sample composition. Separation between the two groups was limited (silhouette coefficient = 0.294), while the correspondence between classifications obtained from unstandardised and Z-standardised variables was minimal (ARI = 0.008), demonstrating a strong influence of scaling on cluster membership. The final classification included 1070 adolescents (55.4%) in the Higher Physical Activity profile and 862 (44.6%) in the Lower Physical Activity profile. The profiles showed no clear differences in school-day sleep duration, television/computer use, or internet-related risk. Weekend sleep duration differed between profiles (V = 0.120, *p* = 0.001). The Higher Physical Activity profile had higher Pro-Healthy Diet Index (r = 0.100, *p* < 0.001) and Non-Healthy Diet Index scores (r = 0.090, *p* < 0.001), slightly higher energy-drink intake (r = 0.080, *p* = 0.001), and more frequent out-of-home meal consumption (V = 0.079, *p* = 0.002). Effect sizes for co-occurring behaviours were generally small. **Conclusions**: Empirically distinct movement-behaviour profiles showed only partial correspondence with broader lifestyle characteristics. Higher physical activity coexisted with both more and less favourable dietary characteristics, whereas the Lower Physical Activity profile did not show a consistently adverse sleep or digital-behaviour pattern. These findings indicate that physical activity level alone should not be treated as a proxy for overall adolescent lifestyle quality. Given the exploratory, cross-sectional design and method-dependent nature of the profiles, the findings should be interpreted as hypothesis-generating rather than as evidence for fixed lifestyle types or profile-specific interventions.

## 1. Introduction

Adolescence is a developmental period during which health-related behaviours, including physical activity, sedentary behaviour, diet, sleep, and digital-media use, become established and may persist into later life [1,2]. These behaviours are relevant to public health because they form broader lifestyle patterns that may be associated with subsequent physical and psychosocial health [1,3]. Lifestyle patterns may emerge early, persist over time, and be socially patterned, making adolescence an important period for examining how health-related behaviours coexist within individuals [2].

Physical activity and sedentary behaviour are increasingly considered within a broader behavioural context rather than as isolated exposures [4,5]. Time allocated to one movement behaviour may coexist with or constrain time available for others, while movement behaviours may also occur alongside dietary, sleep, and other health-related behaviours [4,5,6,7,8]. Previous studies have shown that less favourable behaviours can co-occur during childhood and adolescence, with combinations involving low physical activity, sedentary or screen-based behaviours, and less favourable dietary patterns commonly reported [6]. However, the presence of one favourable or unfavourable behaviour does not necessarily indicate a similarly favourable or unfavourable pattern across other lifestyle domains.

Indeed, adolescent behavioural profiles are not uniformly healthy or unhealthy [9,10,11]. High physical activity may coexist with less favourable dietary choices, greater energy-drink consumption, high screen exposure, or other risk-related behaviours [9,10]. Conversely, lower sedentary exposure does not necessarily imply favourable behaviour across all other domains [11]. Such mixed configurations are important because they challenge the assumption that a single movement behaviour can serve as a proxy for an adolescent’s broader lifestyle pattern. Accordingly, examining physical activity and sedentary behaviour together with co-occurring behaviours may provide a more nuanced description of adolescent lifestyle than analysing these behaviours separately.

Traditional variable-oriented approaches are well suited to estimating associations between individual behaviours and selected outcomes, but they do not directly identify subgroups of individuals characterised by similar combinations of behaviours [12]. Person-oriented methods, including cluster analysis and latent class or profile approaches, provide a complementary strategy by identifying empirically derived groups with similar behavioural configurations [12,13]. In adolescent lifestyle research, such approaches have identified groups differing in physical activity, sedentary behaviour, sleep, screen-related behaviours, and diet [4,5,9,11]. These empirical profiles should not be interpreted as fixed or inherent adolescent types; rather, they represent sample- and method-dependent behavioural configurations that can be useful for describing heterogeneity within a population.

Several studies illustrate the value of this approach. Alosaimi et al. identified healthy, mixed, and unhealthy clusters based on diet, physical activity, screen time, and sleep and examined their associations with subsequent changes in adiposity indicators [9]. Mayne et al. demonstrated the co-occurrence of multiple unhealthy behaviours among children and adolescents [6], while Jonsson et al. showed that adolescent health and lifestyle behaviours form distinct patterns that vary according to demographic and sociodemographic characteristics [11]. Faria et al. identified behavioural classes related to physical activity and sedentary behaviour among Brazilian adolescents [5], and Costa et al. reported 24 h movement-behaviour classes based on physical activity, screen time, and sleep that were associated with cardiorespiratory fitness [4]. Collectively, these studies demonstrate that person-oriented analyses can reveal behavioural heterogeneity that may be obscured when individual behaviours are considered separately, while also showing that the identified profiles depend on the population studied, the behaviours included, and the analytical approach used.

Despite this growing literature, several gaps remain. First, many person-oriented studies derive broad lifestyle profiles by including movement, dietary, sleep, and digital behaviours simultaneously, whereas fewer studies first derive empirical profiles specifically from physical activity and sedentary behaviour and subsequently examine how these movement-behaviour profiles differ across other lifestyle domains [5,14]. This distinction allows co-occurring behaviours to be evaluated independently of the variables used to define the profiles. Second, previous studies have often focused on a limited combination of diet, sleep, or screen-related measures, while fewer have jointly examined dietary indices, between-meal snacking, out-of-home meal consumption, energy-drink intake, television/computer use, internet-related risk, and broader health-behaviour domains as characteristics co-occurring with movement-behaviour profiles [6,11]. Third, mixed lifestyle configurations remain insufficiently characterised, particularly the possibility that higher physical activity may coexist with both favourable and less favourable behaviours [9,10].

The present study was designed to address these gaps by deriving empirical movement-behaviour profiles exclusively from physical activity and sedentary behaviour variables, and then comparing the resulting profiles across a broad set of co-occurring dietary, sleep, television/computer-use, internet-related, and general health behaviours. This approach separates the behaviours used to construct the profiles from those subsequently used to characterise them and allows examination of whether differences in movement behaviour correspond to consistently favourable or unfavourable patterns across other lifestyle domains.

Accordingly, the aim of this study was to identify empirical physical activity–sedentary behaviour profiles among adolescents and to examine whether these profiles differed in selected co-occurring lifestyle behaviours. We hypothesised that empirically derived movement-behaviour profiles would differ across at least some dietary, sleep, digital, and general health-behaviour domains. Given the exploratory and person-oriented nature of the analysis, no directional hypothesis was specified for individual co-occurring behaviours.

## 2. Materials and Methods

### 2.1. Study Design and Setting

This cross-sectional, school-based observational study was conducted among adolescents attending secondary schools in the Świętokrzyskie Voivodeship in south-central Poland during the 2024/2025 school year. The study formed part of the quantitative component of the project “Youth Lifestyles and Their Health Implications: Conclusions for Pedagogical and Psychological Support and Risk Prevention” (Polish title: “Style życia młodzieży i ich zdrowotne implikacje. Wnioski w obszarze pedagogiczno-psychologicznego wsparcia oraz profilaktyki zagrożeń”), implemented by the Jan Kochanowski University in Kielce, Poland.

A related analysis based on the same source project has subsequently been published and addressed a distinct research question using a different analytical strategy [15].

The project was co-financed from the state budget under the Polish Ministry of Education and Science programme “Science for Society II” (project No. NdS-II/SP/0429/2023/01; funding amount and total project value: PLN 1,029,600.00). Reporting follows the Strengthening the Reporting of Observational Studies in Epidemiology (STROBE) recommendations for cross-sectional studies.

### 2.2. Participants and Sampling

The target population comprised second-year students aged 15–17 years attending general secondary schools, technical secondary schools, and stage I vocational schools in the Świętokrzyskie Voivodeship. The sampling frame was constructed using the Polish Educational Information System (System Informacji Oświatowej, SIO) and updated using the register of educational institutions maintained by the Regional Board of Education in Kielce.

The initial sampling frame comprised 253 upper-secondary schools. Schools for adults, schools with an estimated enrolment of fewer than 10 students, and vocational preparation schools intended for young people with multiple disabilities or moderate-to-severe intellectual disabilities were excluded (*n* = 25). Schools were subsequently selected using stratified random sampling according to school type and location. The location strata comprised large cities (>100,000 inhabitants), medium-sized urban areas, and small towns, whereas the school-type strata comprised general secondary, technical secondary, and stage I vocational schools. A total of 53 schools were randomly selected. When a selected school declined participation, another school was randomly selected from the corresponding stratum.

The source project targeted approximately 3500 students from an estimated regional population of approximately 12,500 students in the relevant secondary-school cohort. The resulting project dataset comprised 3307 adolescents, including 1727 girls and 1580 boys, with a mean age of 15.89 ± 0.71 years and an age range of 15–17 years.

The present person-oriented analysis used all observations available in the source project dataset that met the analytical eligibility criteria. Eligibility for profile derivation required complete and analytically valid values for each of the four movement-behaviour variables used in the clustering procedure after final IPAQ-LF data processing. This resulted in a primary analytical sample of 1932 adolescents. The remaining 1375 records were not included in profile derivation because one or more of the required IPAQ-LF variables were missing, incomplete, internally inconsistent, or did not meet the final analytical eligibility criteria.

### 2.3. Data Collection

Data were collected in participating schools using anonymous, self-administered paper questionnaires. The survey package included standardised instruments and project-specific questions. Data used in the present analysis were obtained from the International Physical Activity Questionnaire–Long Form (IPAQ-LF), the KomPAN Dietary Habits and Nutrition Beliefs Questionnaire, the Problematic Internet Use Test (TPUI-22), the Health Behaviour Inventory (HBI), and questions concerning sociodemographic and anthropometric characteristics.

The reference period for physical activity and sedentary behaviour was the preceding seven days. No separate subsample was selected specifically for the present cluster analysis; all records from the source dataset that fulfilled the final analytical eligibility criteria were considered.

### 2.4. Ethical Considerations

The project was approved by the Research Ethics Committee of the Faculty of Pedagogy and Psychology, Jan Kochanowski University in Kielce (Resolution No. 5/2023 of 21 December 2023). Participation was voluntary. As the study population comprised adolescents aged 15–17 years, written informed consent was obtained from a parent or legal guardian, and the adolescents provided assent.

Students were informed about the purpose and general procedures of the study, the confidential nature of their responses, their right to decline participation, and their right to withdraw during questionnaire completion without any adverse consequences. The questionnaires contained no direct personal identifiers, and results were reported only in aggregate form. Paper questionnaires and consent documentation were stored separately in secured university facilities. Electronic data were stored in password-protected files or university systems accessible only to authorised members of the research team.

### 2.5. Sociodemographic and Anthropometric Variables

Sociodemographic variables included sex, age, place of residence, school type, and maternal and paternal education. Place of residence was classified as rural area, small/medium-sized town, or large city. Maternal and paternal education were recorded separately and classified as elementary/vocational, secondary, or university education.

Participants self-reported body height and body mass. Body mass index (BMI) was calculated as body mass in kilograms divided by height in metres squared (kg/m^2^). Sociodemographic and anthropometric variables were used to characterise the retained movement-behaviour profiles.

Because recruitment was restricted to second-year secondary-school students, the study population had a narrow age distribution of 15–17 years.

### 2.6. Physical Activity and Sedentary Behaviour

Physical activity and sitting time were assessed using the IPAQ-LF. The questionnaire assesses walking and moderate- and vigorous-intensity physical activity across work or study, transportation, domestic and gardening, and leisure-time domains during the preceding seven days. It also assesses sitting time on weekdays and weekend days.

Before clustering, IPAQ-LF responses were subjected to the final data-quality screening and processing procedure. Reported durations of walking, moderate-intensity physical activity, and vigorous-intensity physical activity exceeding 180 min/day were truncated to 180 min/day. Responses indicating no activity in a given component were coded as zero when consistent with the questionnaire response logic. Responses for which a valid weekly value could not be derived because of missing or internally inconsistent frequency or duration information were treated as missing. No mean-value imputation was used for variables entering the clustering analysis.

For each physical-activity item, the reported number of minutes per day was multiplied by the number of days per week, and values were summed across the relevant IPAQ-LF domains. Walking, moderate-intensity physical activity, and vigorous-intensity physical activity were expressed in minutes per week. Weekly sedentary time was calculated as five times the reported weekday sitting time plus two times the reported weekend-day sitting time and was also expressed in minutes per week.

Only participants with complete, analytically eligible weekly values for sedentary time, walking, moderate-intensity physical activity, and vigorous-intensity physical activity were included in profile derivation.

### 2.7. Sleep Duration and Television/Computer Use

Sleep duration was assessed using two KomPAN questionnaire items concerning average daily sleep duration on school days and on days off from school. Sleep duration was analysed separately for school days and weekend days and classified as <7 h/day, 7–8 h/day, or ≥9 h/day. These categories were treated descriptively. In particular, ≥9 h/day of weekend sleep was not prespecified as an unfavourable category.

Television/computer use was assessed using a KomPAN item asking participants how many hours per day they usually spent watching television or using a computer. Responses were classified as <2 h/day, ≥2 to <4 h/day, ≥4 to <6 h/day, or ≥6 h/day.

Because the item did not comprehensively capture smartphone, tablet, gaming-console, or other mobile-device exposure, it is referred to as television/computer use rather than total screen time in the revised analysis.

### 2.8. Internet-Related Risk

Internet-related risk was assessed using the TPUI-22, the Polish adaptation of Young’s Internet Addiction Test. The instrument contains 22 items rated from 0 (“never”) to 5 (“always”), yielding a total score from 0 to 110.

Scores were classified as very low (0–1 points), low (2–10 points), moderate (11–49 points), high (50–79 points), or very high (80–110 points). The very-low and low categories were combined for between-profile analyses because of the small number of observations in the lowest categories.

### 2.9. Dietary Behaviours

Dietary behaviours were assessed using the KomPAN questionnaire. Between-meal snacking and eating meals away from home were recorded using the following frequency categories: never, 1–3 times/month, once/week, several times/week, once/day, and several times/day.

For between-profile comparisons, each variable was collapsed into three categories: never or 1–3 times/month, once or several times/week, and once or several times/day. Energy-drink consumption was converted to daily frequency using the KomPAN conversion factors and analysed as a continuous frequency-per-day variable.

The Pro-Healthy Diet Index (pHDI-10) and Non-Healthy Diet Index (nHDI-14) were calculated according to the KomPAN methodology. Reported consumption frequencies were converted into daily equivalents using factors of 0 for never, 0.06 for 1–3 times/month, 0.14 for once/week, 0.50 for several times/week, 1 for once/day, and 2 for several times/day.

The pHDI-10 comprised 10 food groups considered potentially beneficial to health: wholemeal bread, coarse groats, milk, fermented milk beverages, cottage cheese, white meat, fish, legumes, fruit, and vegetables. Daily frequencies were summed and converted to a 0–100-point scale by dividing the raw score by 20 and multiplying by 100.

The nHDI-14 comprised 14 food groups considered potentially adverse to health: white bread; refined groats, pasta, and rice; fast food; fried meat- or flour-based dishes; butter; lard; yellow and processed cheese; cold cuts, sausages, and frankfurters; red meat; sweets and confectionery; canned meat products; sugar-sweetened carbonated beverages; energy drinks; and alcoholic beverages. Daily frequencies were summed and converted to a 0–100-point scale by dividing the raw score by 28 and multiplying by 100.

Higher pHDI-10 and nHDI-14 scores indicate more frequent consumption of foods represented in the respective indices. The two indices were therefore interpreted as separate dimensions and not as opposite ends of a single dietary-quality continuum.

### 2.10. Health Behaviour Inventory

General health behaviours were assessed using the Health Behaviour Inventory. The instrument comprises 24 items rated from 1 (“almost never”) to 5 (“almost always”) and covers four domains: nutritional behaviours, preventive behaviours, positive mental attitude, and health practices.

Each domain contains six items, producing domain scores ranging from 6 to 30. Higher scores indicate more frequent self-reported health-promoting behaviours. All four domain scores were examined as co-occurring behavioural characteristics in the revised analysis.

### 2.11. Derivation and Internal Validation of Movement-Behaviour Profiles

Empirical movement-behaviour profiles were derived using k-means clustering. The four input variables were weekly sedentary time, walking physical activity, moderate-intensity physical activity, and vigorous-intensity physical activity.

Because the four variables differed substantially in dispersion and k-means clustering is based on Euclidean distances, each variable was Z-standardised before clustering. This placed the four movement-behaviour dimensions on a comparable scale and reduced the possibility that cluster allocation would be disproportionately driven by the variable with the greatest numerical dispersion.

Candidate solutions ranging from two to six clusters (k = 2–6) were evaluated. Initial cluster centres were selected randomly, and 100 initialisations were used to reduce dependence on a particular starting configuration.

Internal validity was evaluated using the average silhouette coefficient, Calinski–Harabasz index, and Davies–Bouldin index. Stability across repeated clustering solutions was assessed using the Adjusted Rand Index (ARI). The number of clusters was selected using the combined evidence from separation, compactness, stability, parsimony, and substantive interpretability rather than on the basis of a single validation index. ARI was calculated by comparing membership assignments from k-means runs initiated with different random centroid configurations while keeping the participant dataset unchanged. Accordingly, this analysis evaluated repeatability in relation to algorithm initialisation only and provides no information about the behaviour of the clustering solution after resampling participants. Bootstrap and subsampling procedures were not undertaken. On this basis, the two-cluster solution was retained for the analysis. The profiles were labelled Higher Physical Activity (Higher PA) and Lower Physical Activity (Lower PA) after inspection of the cluster-specific values on the original minute-per-week scale.

A processing sensitivity analysis repeated the two-cluster procedure using the original unstandardised minute-per-week values. Agreement between the raw-data and standardised solutions was quantified using the ARI. This analysis was used to assess the sensitivity of cluster membership to variable scaling and whether variables with greater dispersion disproportionately influenced the unstandardised solution.

### 2.12. Statistical Analysis

Categorical variables were summarised as numbers and percentages. Continuous variables were summarised using means and standard deviations and, where informative for non-normal distributions, medians and interquartile ranges. Percentages in profile-specific analyses were calculated using the number of valid observations within each profile.

Because the retained solution comprised two profiles, categorical outcomes were compared using Pearson’s chi-square test, whereas continuous outcomes were compared using the Mann–Whitney U test.

Effect-size estimates were reported alongside *p*-values. The phi coefficient (φ) was used for 2 × 2 categorical comparisons, Cramér’s V for categorical tables with more than two categories, and the rank-based effect size r for continuous comparisons.

The four movement-behaviour variables used to derive the profiles were presented descriptively and were not subjected to inferential interpretation as independent validation of the clustering solution.

Comparisons of sleep, television/computer use, internet-related risk, dietary variables, and Health Behaviour Inventory domains were considered exploratory. No global multiplicity correction was applied. Consequently, raw *p*-values were interpreted together with effect-size estimates and the consistency of the overall pattern rather than as confirmatory evidence in isolation. All tests were two-sided, with *p* < 0.05 used as a conventional threshold for statistical significance.

The principal clustering, descriptive, and inferential analyses were performed using TIBCO Statistica version 13.3 (TIBCO Software Inc., Palo Alto, CA, USA). Internal-validation and ARI calculations were obtained from the final analytical dataset using the standard definitions of the respective indices.

### 2.13. Missing Data and Analysis Population

Missing data were handled differently for variables used to derive the movement-behaviour profiles and for variables used only in between-profile comparisons.

For clustering, a complete-case approach was applied to the four IPAQ-LF-derived input variables after final data cleaning and processing. No missing-value imputation was performed. This resulted in a primary clustering sample of 1932 adolescents from the original project dataset of 3307 participants.

For sociodemographic, anthropometric, sleep, television/computer-use, internet-related, dietary, and HBI variables, no imputation was performed. Descriptive analyses and between-profile comparisons used all available observations for the relevant variable; consequently, denominators could vary between outcomes.

Valid and missing observations for selected variables are reported in the Supplementary Materials. The substantial reduction from the original dataset to the clustering sample was considered a potential source of selection bias and was taken into account when interpreting the findings.

Consequently, the resulting movement-behaviour profiles apply specifically to participants for whom all four IPAQ-LF variables required for clustering were complete and analytically valid. They should not be regarded as profiles representing the entire original project cohort.

## 3. Results

### 3.1. Analytical Sample

The original study sample comprised 3307 adolescents. Following the final cleaning and processing of the IPAQ-LF variables used for profile derivation, 1932 participants had complete and analytically eligible data for weekly sedentary time, walking, moderate-intensity physical activity, and vigorous-intensity physical activity. These participants constituted the primary analytical sample. A total of 1375 participants (41.58% of the original sample) were not included in the clustering analysis because their physical activity or sedentary behaviour data did not meet the requirements of the final analytical dataset. Sex information was available for both the included and excluded participants. Girls accounted for 51.0% of the analytical sample (986/1932) and 53.9% of those not included in profile derivation (741/1375), indicating only a small difference in sex distribution between the two groups.

The retained two-profile solution comprised 1070 adolescents (55.4%) in the Higher Physical Activity (Higher PA) profile and 862 adolescents (44.6%) in the Lower Physical Activity (Lower PA) profile. Comparisons of variables not used to derive the profiles were conducted using available observations; therefore, denominators varied across outcomes. Profile-specific valid and missing observations for selected variables are reported in Supplementary Table S4.

### 3.2. Selection, Internal Validation, and Sensitivity of the Cluster Solution

K-means solutions ranging from two to six clusters were evaluated using the silhouette coefficient, Calinski–Harabasz index, Davies–Bouldin index, and Adjusted Rand Index (ARI) as a measure of stability. Among the candidate solutions, k = 2 produced a silhouette coefficient of 0.294, a Calinski–Harabasz index of 880.20, and near-complete agreement between classifications generated from repeated random starts (ARI = 0.999). The Davies–Bouldin index followed a different pattern, decreasing as the number of clusters increased and reaching its minimum for k = 6. The two-cluster model was ultimately selected after jointly considering cluster separation, compactness, repeatability across initialisations, parsimony, and substantive interpretability. Validation statistics for all solutions from k = 2 through k = 6 are provided in Supplementary Table S2.

The preprocessing sensitivity analysis showed very low agreement between the two-cluster solution obtained using the original minute-per-week values and the primary solution based on Z-standardised variables (ARI = 0.008). In the analysis based on raw values, cluster allocation was predominantly influenced by sedentary time, whereas standardisation placed the four movement-behaviour dimensions on a comparable scale. The Z-standardised two-profile solution was therefore retained as the primary analytical solution (Supplementary Table S3).

### 3.3. Movement-Behaviour, Sociodemographic, and Anthropometric Characteristics of the Profiles

The Higher PA profile had higher mean levels of vigorous, moderate, and walking physical activity and lower mean sedentary time than the Lower PA profile (Table 1). Because these four movement-behaviour variables were used directly to derive the profiles, they are presented descriptively and were not subjected to inferential interpretation as independent evidence of between-profile differences.

Sociodemographic and anthropometric differences between the profiles were generally small. The Higher PA profile included a lower proportion of girls than the Lower PA profile (46.64% vs. 56.50%; φ = 0.100, *p* = 0.001). BMI was slightly higher in the Higher PA profile (r = 0.080, *p* = 0.001). School-type distribution did not differ clearly between the profiles (V = 0.055, *p* = 0.064). Small between-profile differences were observed for place of residence (V = 0.070, *p* = 0.021) and paternal education (V = 0.082, *p* = 0.008), whereas maternal education showed no clear difference (V = 0.040, *p* = 0.304). Detailed distributions are presented in Table 1.

### 3.4. Sleep and Digital Behaviours

School-day sleep duration showed no clear between-profile difference (V = 0.053, *p* = 0.073). Weekend sleep duration differed between profiles, with a small effect size (V = 0.120, *p* = 0.001). Sleep duration of ≥9 h/day on weekends was reported by 59.61% of adolescents in the Lower PA profile and 49.90% in the Higher PA profile, whereas <7 h/day was reported by 6.45% and 12.85%, respectively.

Television/computer use did not differ clearly between profiles (V = 0.053, *p* = 0.161). Similarly, the distribution of internet-related risk showed no clear between-profile difference (V = 0.048, *p* = 0.209) (Table 2).

### 3.5. Dietary and General Health Behaviours

Between-meal snacking showed no clear between-profile difference (V = 0.054, *p* = 0.066). Out-of-home meal consumption differed between the profiles, although the effect size was small (V = 0.079, *p* = 0.002). More frequent out-of-home meal consumption was somewhat more common in the Higher PA profile.

The Higher PA profile had higher Pro-Healthy Diet Index scores (r = 0.100, *p* < 0.001) and higher Non-Healthy Diet Index scores (r = 0.090, *p* < 0.001) than the Lower PA profile. Energy-drink intake was also slightly higher in the Higher PA profile (r = 0.080, *p* = 0.001). All of these between-profile effect sizes were small.

For the Health Behaviour Inventory, preventive behaviours (r = 0.040, *p* = 0.122), nutritional behaviours (r = 0.040, *p* = 0.058), and positive mental attitude (r = 0.010, *p* = 0.463) showed no clear between-profile differences. Health-practice scores were slightly higher in the Lower PA profile (r = 0.070, *p* < 0.001), with a small effect size (Table 3). Full descriptive statistics for continuous outcomes, including medians and interquartile ranges, are provided in Supplementary Table S5.

## 4. Discussion

### 4.1. Principal Findings

The present study identified two empirical movement-behaviour profiles among 1932 adolescents with complete and analytically eligible IPAQ-LF data: a Higher Physical Activity (Higher PA) profile and a Lower Physical Activity (Lower PA) profile. The final two-profile classification was highly consistent across alternative random starting configurations (ARI = 0.999), whereas separation of the two groups remained modest (silhouette coefficient = 0.294). The marked differences in physical activity and sedentary time between the profiles are expected because these variables directly determined cluster membership and therefore should be regarded as defining features of the profiles rather than independent study outcomes. More importantly, the hypothesis that the resulting movement-behaviour profiles would differ across co-occurring lifestyle domains was only partially supported. Clear between-profile differences were not observed for school-day sleep duration, television/computer use, internet-related risk, or most Health Behaviour Inventory domains, whereas differences were detected for weekend sleep duration and selected dietary characteristics. Where differences in co-occurring behaviours were present, their effect sizes were generally small.

This pattern does not support a simple distinction between globally favourable and unfavourable adolescent lifestyle profiles. Instead, the Higher PA profile combined higher levels of walking, moderate-intensity and vigorous-intensity physical activity with both more frequent consumption of foods included in the Pro-Healthy Diet Index and more frequent consumption of foods included in the Non-Healthy Diet Index, together with slightly higher energy-drink intake and somewhat more frequent out-of-home meal consumption. Conversely, the Lower PA profile did not show a consistently less favourable pattern across sleep and digital-behaviour domains. These findings are consistent with previous person-oriented research showing that health-related behaviours in children and adolescents often occur in heterogeneous combinations rather than along a single healthy–unhealthy continuum [9,11,12,16,17]. Systematic reviews of activity-related typologies have similarly demonstrated substantial heterogeneity in the behavioural combinations observed across youth populations and have identified active, inactive, sedentary, and mixed configurations rather than one reproducible profile structure [16,17].

The present findings extend person-oriented research in two ways. First, the movement-behaviour profiles were derived exclusively from physical activity and sedentary behaviour variables, while sleep, digital, dietary, and general health behaviours were examined subsequently as co-occurring characteristics rather than as components of the clustering solution. Second, the results indicate that even clearly differentiated movement-behaviour profiles may correspond only partially to broader lifestyle differences. This interpretation is compatible with evidence that combinations of physical activity, sedentary behaviour, and sleep are heterogeneous across children and adolescents and that favourable levels of one movement behaviour do not necessarily imply favourable levels of all others [18]. Consequently, the principal contribution of the present study is not the identification of fixed “healthy” and “unhealthy” adolescent types, but rather the demonstration that empirically distinct movement-behaviour profiles show only limited correspondence with broader lifestyle characteristics.

### 4.2. Movement-Behaviour Profiles, Sleep, and Digital Behaviours

The two movement-behaviour profiles showed limited differentiation in sleep and digital behaviours. School-day sleep duration did not differ clearly between the Higher PA and Lower PA profiles, and neither television/computer use nor internet-related risk showed meaningful between-profile differences. Weekend sleep duration differed statistically between profiles, but the effect size was small (V = 0.120). Adolescents in the Lower PA profile more often reported ≥9 h/day of sleep on weekends, whereas <7 h/day was more common in the Higher PA profile. This pattern should not be interpreted as evidence that the Lower PA profile had a less favourable sleep profile. Longer weekend sleep may reflect adequate sleep duration, recovery from weekday sleep restriction, or differences in sleep timing; however, bedtime, wake time, sleep regularity, and social jetlag were not measured in the present study. Accordingly, the weekend-sleep finding should be interpreted descriptively rather than classified as favourable or unfavourable.

Previous studies have reported links between physical activity, sedentary behaviour, screen exposure, and sleep, but the strength and consistency of these associations vary across populations and analytical approaches. In a latent class analysis of US adolescents, combinations of physical activity and screen-based sedentary behaviour were associated with sleep duration, with the high physical activity/low screen-based sedentary subgroup showing the most favourable probability of sufficient sleep [19]. Similarly, person-oriented studies have demonstrated that physical activity, screen use, and sleep can form distinct behavioural configurations in adolescents. Sanz-Martín et al. identified three clusters based on moderate-to-vigorous physical activity, screen time, and sleep among 663 Spanish adolescents, with only a minority meeting recommendations for all three behaviours simultaneously [20]. Longitudinal person-oriented evidence also indicates that activity and screen behaviours may combine in several distinct ways. Parker et al. identified “Actives”, “Inactives”, and “Sedentary gamers” among older adolescents, with substantial stability in profile membership over two years [21].

At the same time, the absence of clear sleep and digital differences in the present study is compatible with evidence that these behaviours do not necessarily covary strongly with physical activity. A systematic review and meta-analysis of 11 studies including 9622 children and youth found only a weak association between sedentary time and subsequent-night sleep duration, while positive physical activity–sleep associations were observed mainly in studies adjusting for accelerometer wear time [22]. More recent longitudinal data from adolescents similarly found no relationship between moderate-to-vigorous physical activity and the measured sleep variables and did not support a reciprocal physical activity–sleep relationship [23]. These findings suggest that movement behaviours, sleep, and digital engagement may interact, but they should not be expected to form a single coherent behavioural gradient in every adolescent population.

The interpretation of digital behaviours also requires consideration of how they were measured. Previous systematic reviews have linked higher screen exposure with shorter sleep duration and poorer sleep outcomes in children and adolescents [24,25], while problematic internet use has been associated with sleep problems [26]. Social-media engagement and nighttime technology use have also been related to sleep timing and social-jetlag indicators [27,28,29]. However, these findings should not be directly extrapolated to the present television/computer-use measure. The KomPAN item assessed time spent watching television or using a computer and did not comprehensively capture smartphone, tablet, gaming-console, or other mobile-device exposure. The lack of a between-profile difference therefore indicates no clear difference in the measured television/computer-use behaviour, rather than equivalence in total contemporary screen exposure. Similarly, the absence of a difference in internet-related risk does not exclude differences in specific forms, timing, or contexts of digital-media use that were not measured.

Overall, the sleep and digital results do not support the interpretation that the Lower PA profile represented a broader adverse sleep–digital lifestyle pattern. Rather, they suggest that movement-behaviour differences were only partly accompanied by differences in adjacent behavioural domains. This distinction is important because it argues against treating lower physical activity or higher sedentary behaviour as a proxy for adverse sleep or digital behaviour and reinforces the need to interpret empirical movement-behaviour profiles as domain-specific configurations rather than comprehensive lifestyle types.

### 4.3. Higher Physical Activity and Co-Occurring Dietary Behaviours

The clearest differences in behaviours not used to derive the movement profiles were observed in the dietary domain, although their magnitude was small. Adolescents in the Higher PA profile had higher Pro-Healthy Diet Index scores than those in the Lower PA profile (r = 0.100), but they also had higher Non-Healthy Diet Index scores (r = 0.090), slightly higher energy-drink intake (r = 0.080), and somewhat more frequent out-of-home meal consumption (V = 0.079). These findings should not be interpreted as contradictory because the pHDI-10 and nHDI-14 represent separate dimensions of food-consumption frequency rather than opposite ends of a single dietary-quality scale. Accordingly, higher scores on both indices indicate that adolescents in the Higher PA profile reported more frequent consumption of foods contributing to both the pro-healthy and non-healthy indices. Taken together, the results suggest that higher physical activity did not correspond to a uniformly favourable dietary pattern.

This interpretation is consistent with person-oriented research showing that physical activity and dietary behaviours do not necessarily align along a simple healthy–unhealthy continuum. Reviews of behavioural clustering in young people have repeatedly identified heterogeneous and mixed combinations of activity, sedentary behaviour, and diet [16,17]. Alosaimi et al. likewise identified healthy, unhealthy, and mixed adolescent lifestyle clusters based on diet, physical activity, screen time, and sleep [9]. A systematic review focusing specifically on children and adolescents aged 11–16 years also concluded that health-related behaviours frequently cluster in combinations that include both favourable and unfavourable components rather than forming consistently concordant behavioural profiles [30]. Polish data are also relevant in this context. Wądołowska et al. identified distinct dietary–lifestyle patterns among Polish teenagers, demonstrating that physical activity and dietary behaviours combine into multidimensional configurations rather than representing interchangeable indicators of an overall healthy lifestyle [31].

Energy-drink intake provides a particularly relevant example of this behavioural complexity. In the present study, adolescents in the Higher PA profile reported slightly more frequent energy-drink consumption, although the effect size was small. Previous Polish research has shown that energy-drink consumption is common among physically active adolescents and may coexist with participation in organised sport [32]. Similarly, Larson et al. reported that sports- and energy-drink consumption among US adolescents was associated with higher physical activity but also with less favourable behaviours, including unhealthy beverage consumption, cigarette smoking, and greater screen-media use [33]. A systematic review found that energy-drink consumption in children and adolescents was associated with a range of behavioural and lifestyle correlates, including poorer dietary habits and substance-related behaviours, while associations with sport and physical activity were heterogeneous [34]. Australian data further showed that energy-drink intake clustered with unhealthy dietary behaviours and shorter sleep duration [35]. These studies support the interpretation that energy-drink use should not be considered inconsistent with high physical activity; rather, the two behaviours may coexist within the same adolescent lifestyle pattern.

The difference in out-of-home meal consumption should be interpreted more cautiously. More frequent out-of-home eating was somewhat more common in the Higher PA profile, but the present study did not assess the type of food consumed, nutritional composition of the meals, reasons for eating outside the home, or whether meals were related to school, sport participation, peer activities, or family routines. Consequently, out-of-home meal consumption cannot be classified as intrinsically favourable or unfavourable on the basis of these data. Recent observational evidence also indicates that adolescent physical activity and dietary behaviours may change independently and that meeting recommendations in one behavioural domain does not imply adherence in others [36]. The present finding should therefore be regarded primarily as evidence of behavioural heterogeneity rather than as an indicator of poorer dietary quality.

Importantly, the observed dietary differences were small and were derived from unadjusted comparisons. The Higher PA and Lower PA profiles also differed modestly in sex distribution, BMI, place of residence, and paternal education. These characteristics may themselves be associated with both physical activity and dietary behaviour [11,31]. Because the final analysis did not include multivariable adjustment, it cannot be determined whether the dietary differences observed between profiles reflect profile membership independently of sociodemographic composition. Therefore, the present results should not be interpreted as showing that higher physical activity causes greater consumption of energy drinks, non-healthy foods, or meals outside the home. Rather, they demonstrate that higher physical activity can coexist with both more and less favourable dietary characteristics and reinforce the broader conclusion that physical activity alone should not be used as a proxy for overall lifestyle quality.

### 4.4. Methodological Robustness and Sensitivity of the Movement-Behaviour Profiles

To limit reliance on a subjectively chosen cluster structure, we compared solutions containing two to six clusters using several internal-validation measures and the consistency of classifications obtained from repeated random starts. For k = 2, the silhouette coefficient was 0.294, and the Calinski–Harabasz index was 880.20, both the highest among the evaluated solutions; this solution also showed almost complete agreement across initialisations (ARI = 0.999). At the same time, the silhouette value indicates only modest cluster separation and should not be interpreted as evidence of a strongly separated underlying population structure. The Davies–Bouldin index also decreased for more complex solutions, illustrating that different validation indices may favour different values of k. The final choice of the two-cluster solution therefore reflected the combined evidence from separation, compactness, stability, parsimony, and interpretability rather than reliance on a single criterion. This approach is consistent with methodological recommendations that the number of clusters should not be determined from one validity statistic alone and that internal indices should be interpreted jointly with the substantive structure of the data [37,38].

An ARI of 0.999 across alternative random starts indicates that changing the initial centroids had virtually no effect on the resulting two-group classification for these participants. This should not, however, be interpreted as evidence that the population contains two intrinsically distinct groups or that an equivalent partition would necessarily emerge in another sample. Evaluating reproducibility under changes in sample composition would require resampling approaches such as bootstrap or subsampling, which were beyond the analyses performed in this study. Methodological work has repeatedly shown that initialisation strategy and repeated runs are important for obtaining reproducible k-means solutions [39,40]. Applied lifestyle research has similarly used multiple starting seeds and stability checks to reduce dependence on a particular initial solution [41]. In the present analysis, the use of 100 random initialisations and the near-perfect stability estimate therefore strengthens confidence that the retained partition was not simply a consequence of an arbitrary starting configuration.

By contrast, the preprocessing sensitivity analysis revealed substantial dependence of cluster membership on variable scaling. Agreement between the two-cluster solution derived from raw minute-per-week values and the primary Z-standardised solution was extremely low (ARI = 0.008). In the unstandardised analysis, sedentary time disproportionately influenced Euclidean distances because of its greater dispersion, whereas z-standardisation placed sedentary time, walking, moderate-intensity physical activity, and vigorous-intensity physical activity on a comparable scale. This finding demonstrates that preprocessing was not a trivial analytical step but materially affected the resulting behavioural classification. Such sensitivity is expected in distance-based methods because variables with larger numerical variance can dominate cluster allocation if entered without scaling. Earlier behavioural clustering studies have therefore standardised movement variables before k-means analysis to place them on a common metric and reduce the influence of unequal dispersion [42]. More generally, normalisation has been shown to materially influence cluster quality and reproducibility in biomedical data [43].

The high sensitivity to scaling also places an important limit on the interpretation of the identified profiles. Although the Higher PA and Lower PA solution was highly stable once the Z-standardised preprocessing strategy had been specified, it should not be regarded as evidence of two fixed or naturally occurring adolescent types. Cluster solutions are inherently conditional on the variables entered, their preprocessing, the clustering algorithm, and the criteria used to determine k [12,16,17]. Recent work using activity-pattern data has likewise shown that preprocessing and validation choices can materially alter the apparent robustness and preferred number of clusters [44]. The two groups should therefore be viewed as data-driven configurations specific to the present sample, variables, preprocessing choices, and analytical procedure, rather than as stable adolescent categories expected to generalise universally.

### 4.5. Contribution to the Existing Literature

The present study contributes to the person-oriented literature on adolescent health behaviours by addressing the three gaps identified in the Introduction. First, the movement-behaviour profiles were derived exclusively from physical activity and sedentary behaviour variables, whereas sleep, television/computer use, internet-related risk, dietary behaviours, and general health behaviours were examined subsequently as co-occurring characteristics. This distinction is methodologically important because many previous person-oriented studies have incorporated several lifestyle domains simultaneously when constructing clusters or latent classes [9,11,12,16,17]. Such approaches are valuable for identifying broad lifestyle configurations, but they do not allow behaviours included in the clustering solution to be evaluated independently as external characteristics of the resulting groups. By separating profile-defining from profile-characterising variables, the present study provides a more direct assessment of the extent to which empirically derived movement-behaviour differences correspond to differences in other lifestyle domains.

Second, the study extends previous work by examining a comparatively broad range of co-occurring behaviours within the same analytical framework. Previous reviews have shown that adolescent behavioural clustering studies frequently include physical activity, sedentary behaviour, diet, sleep, or screen-related behaviours, but the specific combinations of variables differ substantially across studies [12,16,17]. Whitaker et al. similarly concluded that studies of children and adolescents aged 11–16 years identify multiple co-occurring health-related behaviours, but that methodological heterogeneity and differences in the behavioural domains included limit the consistency of the resulting clusters [30]. In the present study, sleep duration, television/computer use, internet-related risk, between-meal snacking, out-of-home meal consumption, pro-healthy and non-healthy dietary indices, energy-drink intake, and four Health Behaviour Inventory domains were examined as characteristics external to the clustering model. This broader characterisation allowed the degree of correspondence between movement behaviour and other lifestyle domains to be evaluated rather than assumed.

Third, the findings provide evidence that differences in movement behaviour do not necessarily translate into equally distinct differences across the broader lifestyle pattern. The Higher PA and Lower PA profiles were clearly differentiated by the variables used to derive them, yet their correspondence with co-occurring behaviours was selective and generally weak. In particular, school-day sleep duration, television/computer use, internet-related risk, and most general health-behaviour domains showed little evidence of between-profile differences, whereas the dietary domain showed a more heterogeneous pattern in which Higher PA coexisted with both higher pro-healthy and non-healthy dietary indices. This observation is consistent with systematic evidence that combinations of physical activity, sedentary behaviour, and sleep among young people are heterogeneous and that favourable levels in one behavioural domain do not ensure favourable levels in others [18]. It is also consistent with person-oriented research demonstrating healthy, unhealthy, and mixed behavioural configurations in adolescent populations [9,11,16,17].

Accordingly, the principal contribution of the present study is not the identification of globally “healthy” and “unhealthy” adolescent types. Rather, it demonstrates that empirically distinct physical activity–sedentary behaviour profiles show only partial correspondence with broader lifestyle characteristics. This finding supports a more differentiated interpretation of adolescent movement behaviour: physical activity and sedentary behaviour provide important information about one behavioural domain, but they should not be treated as proxies for the quality of the adolescent’s overall lifestyle. The person-oriented approach used here therefore complements, rather than replaces, variable-oriented analyses by illustrating how movement behaviours are embedded within broader but only partly concordant behavioural contexts [12,16,17].

### 4.6. Practical and Public-Health Implications

The present findings have practical relevance primarily because they indicate that physical activity level alone should not be interpreted as a proxy for the quality of an adolescent’s broader lifestyle. International recommendations appropriately emphasise sufficient physical activity and limitation of sedentary behaviour during childhood and adolescence [45]. However, the Higher PA profile in the present study was not consistently accompanied by more favourable characteristics across dietary, sleep, digital, and general health-behaviour domains, while the Lower PA profile did not show a uniformly adverse pattern outside the movement domain. Accordingly, assessment of adolescent health behaviours may benefit from considering physical activity and sedentary behaviour alongside other lifestyle domains rather than assuming that favourable movement behaviour necessarily reflects favourable behaviour more broadly.

This interpretation is compatible with the broader rationale for multidomain health promotion. Previous systematic evidence indicates that interventions addressing multiple lifestyle risk behaviours in adolescents can produce improvements in selected outcomes, although effects are often modest, heterogeneous, and not consistently sustained over time [46]. Similarly, school-based interventions addressing physical activity [47] or food-related behaviours [48], as well as approaches recognising the role of family context in physical activity, sedentary behaviour, and sleep [49], illustrate that adolescent health behaviours are influenced by multiple settings and determinants. The present study does not establish that interventions tailored to empirically derived movement-behaviour profiles are more effective than conventional or population-wide approaches. Rather, the findings suggest that focusing exclusively on physical activity may overlook co-occurring behaviours that could be relevant when designing or evaluating broader health-promotion strategies.

The results should therefore be regarded as hypothesis-generating for future prospective and intervention research. In particular, subsequent studies could examine whether multidomain behavioural assessment provides additional value beyond movement-behaviour measures alone and whether adolescents with different empirical movement profiles respond differently to particular intervention components. Such questions require longitudinal or experimental designs and cannot be resolved from the present cross-sectional analysis. Consequently, the current findings support consideration of co-occurring behaviours in adolescent health research, but they do not justify prescriptive recommendations for profile-specific intervention packages.

### 4.7. Strengths and Limitations

Several strengths of the present study should be acknowledged. First, the analysis was based on a large school-based dataset drawn from adolescents attending different types of secondary schools and recruited through a stratified sampling strategy, which increased heterogeneity in educational and sociodemographic background. Second, the analytical approach clearly separated the variables used to derive the movement-behaviour profiles from the sleep, digital, dietary, sociodemographic, and general health behaviours subsequently used to characterise them. This distinction reduced the risk of circular interpretation and allowed the extent to which movement-behaviour differences corresponded to broader lifestyle differences to be examined directly. Third, the revised clustering procedure incorporated several safeguards recommended for person-oriented analyses, including Z-standardisation of variables, evaluation of candidate solutions from k = 2 to k = 6, 100 random initialisations, multiple internal-validation indices, assessment of cluster stability, and a sensitivity analysis examining the consequences of variable scaling. Methodological literature emphasises that preprocessing, initialisation, and evaluation of alternative cluster solutions are important because k-means results can depend substantially on these analytical decisions [38,39]. Finally, reporting effect-size estimates alongside *p*-values and treating the co-occurring behavioural comparisons as exploratory helped distinguish statistical detectability from the magnitude of observed differences.

Several limitations are equally important. First, the study was cross-sectional; therefore, no temporal or causal relationships can be inferred between movement-behaviour profile membership and the co-occurring lifestyle characteristics. The analyses establish that selected behaviours were distributed differently across empirically derived profiles, but they do not establish whether physical activity or sedentary behaviour influenced sleep, dietary, or digital behaviours, or vice versa. Second, all movement and co-occurring behavioural variables were self-reported. Recall error, misunderstanding of questionnaire items, and social desirability may therefore have affected the estimated levels of physical activity, sedentary behaviour, diet, sleep, and other health-related behaviours. This is particularly relevant for the IPAQ-LF because the four self-reported movement variables determined profile assignment; measurement error in these variables could consequently result in misclassification of individual profile membership.

Third, complete and analytically acceptable values for all four IPAQ-LF variables required for clustering were available for 1932 of the 3307 adolescents in the source cohort. The remaining 1375 participants (41.58%) were not entered into the clustering procedure because at least one required variable was missing, incomplete, internally inconsistent, or otherwise failed the final eligibility criteria. The proportion of girls was broadly comparable in the included and excluded groups (51.0% and 53.9%, respectively). However, the available dataset did not permit a reliable reconstruction of a corresponding comparison for the full set of sociodemographic and anthropometric variables. Selection bias therefore remains possible, and the profiles identified in this study should be considered representative of the eligible analytical subset rather than of the complete source cohort.

Fourth, repeated k-means runs with different initial centroid configurations yielded almost identical classifications within this dataset (ARI = 0.999), but no bootstrap or subsampling analysis was undertaken. The ARI reported here therefore cannot be used to infer the stability of the cluster structure across different samples. Furthermore, the silhouette coefficient of 0.294 indicates only modest separation, and the classification changed markedly when the analysis was repeated without Z-standardisation (ARI between the raw and standardised solutions = 0.008). This pronounced dependence on preprocessing is consistent with methodological evidence showing that normalisation choices can materially affect clustering results and their reproducibility [43]. Fifth, comparisons of co-occurring behaviours were exploratory and unadjusted. No multivariable models were available to determine whether observed differences were independent of sex, BMI, school type, place of residence, parental education, or other potential confounders. This is relevant because the two profiles differed modestly in several sociodemographic and anthropometric characteristics, and previous person-oriented studies have shown that behavioural profiles can vary according to sex and socioeconomic context. In addition, no global correction for multiple testing was applied. Although interpretation was based on effect sizes and pattern consistency rather than *p*-values alone, the number of comparisons increases the possibility of chance findings. The generally small effect sizes observed outside the movement variables should therefore be given greater weight than statistical significance alone.

Sixth, the measures of sleep and digital behaviour had limited scope. Sleep was assessed as duration categories for school days and weekends, but bedtime, wake time, sleep regularity, sleep quality, and social jetlag were not measured. Consequently, longer weekend sleep cannot be interpreted as either favourable or unfavourable without additional information. Similarly, the KomPAN item assessed television viewing and computer use rather than comprehensive contemporary screen exposure. Smartphone, tablet, gaming-console, and other mobile-device use were not fully captured; therefore, the absence of between-profile differences in television/computer use should not be interpreted as evidence of equivalence in total screen exposure. Previous studies have shown that different forms of screen-based behaviour may have distinct associations with sleep and other health-related outcomes [24,25,26].

Finally, the study did not include objective measures of physical activity, sedentary behaviour, sleep, or digital-media exposure, nor did it assess downstream health outcomes such as cardiorespiratory fitness, adiposity, mental health, self-rated health, or quality of life. Accordingly, the health significance of the identified movement-behaviour profiles cannot be inferred directly from the present data. The sample was also restricted to second-year secondary-school students aged 15–17 years from one Polish region, which limits generalisability to adolescents of other ages, educational settings, geographical regions, or countries. Future research should therefore seek to replicate the profile structure using objective movement measures, broader digital and sleep assessments, prospective designs, and independent populations before stronger conclusions are drawn regarding the stability or health relevance of these empirical movement-behaviour configurations.

## 5. Conclusions

This study identified two empirical movement-behaviour profiles among adolescents: a Higher PA profile and a Lower PA profile. Although the profiles were clearly differentiated by the physical activity and sedentary behaviour variables used to derive them, their correspondence with broader lifestyle characteristics was selective and generally small. The Higher PA profile combined higher physical activity with both more favourable and less favourable dietary characteristics, including slightly higher energy-drink intake, whereas the Lower PA profile did not show a consistently adverse pattern of sleep or digital behaviours.

These findings indicate that movement-behaviour profiles should not be interpreted as globally “healthy” or “unhealthy” adolescent lifestyle types and that physical activity level alone should not be used as a proxy for overall lifestyle quality. Rather, physical activity and sedentary behaviour represent one component of a broader and only partly concordant behavioural context. Given the cross-sectional design, exploratory unadjusted comparisons, and sample- and method-dependent nature of the clustering solution, the findings do not support causal inferences or prescriptive profile-specific interventions. They instead support the value of multidomain behavioural assessment and provide a basis for future prospective studies using objective measures, adjusted analyses, and independent adolescent populations.

## Figures and Tables

**Table 1:** Movement-behaviour, sociodemographic, and anthropometric characteristics of the retained profiles.

Characteristic	Higher PA Profile (*n* = 1070)	Lower PA Profile (*n* = 862)	Effect Size	*p*-Value
Profile-defining movement behaviours, min/week (mean)				
Vigorous physical activity	692.92	285.41	—	—
Moderate physical activity	1124.06	453.62	—	—
Walking	1075.75	533.92	—	—
Sedentary time	2204.82	2367.05	—	—
Sociodemographic and anthropometric characteristics				
Sex			φ = 0.100	0.001
Girls, *n* (%)	499 (46.64)	487 (56.50)		
Boys, *n* (%)	571 (53.36)	375 (43.50)		
BMI, kg/m^2^, mean ± SD	21.82 ± 4.36	21.16 ± 3.47	r = 0.080	0.001
School type			V = 0.055	0.064
Vocational school, *n* (%)	218 (21.63)	155 (18.88)		
General secondary school, *n* (%)	501 (49.70)	453 (55.18)		
Technical school, *n* (%)	289 (28.67)	213 (25.94)		
Place of residence			V = 0.070	0.021
Large city, *n* (%)	129 (15.07)	144 (19.89)		
Small/medium-sized town, *n* (%)	296 (34.58)	218 (30.11)		
Rural area, *n* (%)	431 (50.35)	362 (50.00)		
Father’s education			V = 0.082	0.008
Elementary/vocational, *n* (%)	153 (19.44)	88 (13.37)		
Secondary, *n* (%)	281 (35.71)	258 (39.21)		
University, *n* (%)	353 (44.85)	312 (47.42)		
Mother’s education			V = 0.040	0.304
Elementary/vocational, *n* (%)	173 (21.20)	126 (18.67)		
Secondary, *n* (%)	252 (30.88)	230 (34.07)		
University, *n* (%)	391 (47.92)	319 (47.26)		

Notes: PA, physical activity; SD, standard deviation; φ, phi coefficient; V, Cramér’s V; r, rank-based effect size. Percentages are based on valid responses within each profile. The four movement-behaviour variables were used to derive the profiles and are therefore presented descriptively without inferential interpretation. *p*-values for sociodemographic and anthropometric comparisons are unadjusted.

**Table 2:** Sleep and digital behaviours according to movement-behaviour profile.

Characteristic	Higher PA Profile (*n* = 1070)	Lower PA Profile (*n* = 862)	Effect Size	*p*-Value
School-day sleep duration			V = 0.053	0.073
<7 h/day, *n* (%)	445 (43.80)	364 (44.28)		
7–8 h/day, *n* (%)	512 (50.39)	429 (52.19)		
≥9 h/day, *n* (%)	59 (5.81)	29 (3.53)		
Weekend sleep duration			V = 0.120	0.001
<7 h/day, *n* (%)	130 (12.85)	53 (6.45)		
7–8 h/day, *n* (%)	377 (37.25)	279 (33.94)		
≥9 h/day, *n* (%)	505 (49.90)	490 (59.61)		
Television/computer use			V = 0.053	0.161
<2 h/day, *n* (%)	337 (32.85)	240 (29.09)		
≥2 to <4 h/day, *n* (%)	281 (27.39)	256 (31.03)		
≥4 to <6 h/day, *n* (%)	243 (23.68)	184 (22.30)		
≥6 h/day, *n* (%)	165 (16.08)	145 (17.58)		
Internet-related risk			V = 0.048	0.209
Very low/low, *n* (%)	524 (48.97)	381 (44.20)		
Moderate, *n* (%)	436 (40.75)	389 (45.13)		
High, *n* (%)	98 (9.16)	82 (9.51)		
Very high, *n* (%)	12 (1.12)	10 (1.16)		

Notes: V, Cramér’s V. The KomPAN item assessed television and computer use and should not be interpreted as a comprehensive measure of total contemporary screen exposure. The very-low and low internet-risk categories were combined for analysis. Percentages are based on valid responses. *p*-values are unadjusted exploratory values.

**Table 3:** Dietary and Health Behaviour Inventory characteristics according to movement-behaviour profile.

Characteristic	Higher PA Profile (*n* = 1070)	Lower PA Profile (*n* = 862)	Effect Size	*p*-Value
Between-meal snacking			V = 0.054	0.066
Never/1–3 times per month, *n* (%)	181 (17.30)	115 (13.67)		
Once/several times per week, *n* (%)	428 (40.92)	343 (40.78)		
Once/several times per day, *n* (%)	437 (41.78)	383 (45.54)		
Out-of-home meal consumption			V = 0.079	0.002
Never/1–3 times per month, *n* (%)	577 (53.93)	529 (61.37)		
Once/several times per week, *n* (%)	442 (41.31)	306 (35.50)		
Once/several times per day, *n* (%)	51 (4.77)	27 (3.13)		
Energy-drink intake, times/day, mean ± SD	0.25 ± 0.49	0.18 ± 0.40	r = 0.080	0.001
Pro-Healthy Diet Index, mean ± SD	21.15 ± 11.74	18.71 ± 10.16	r = 0.100	<0.001
Non-Healthy Diet Index, mean ± SD	19.41 ± 11.59	17.32 ± 9.39	r = 0.090	<0.001
HBI preventive behaviours, mean ± SD	17.07 ± 5.04	17.43 ± 4.47	r = 0.040	0.122
HBI nutritional behaviours, mean ± SD	17.70 ± 4.83	17.37 ± 4.47	r = 0.040	0.058
HBI positive mental attitude, mean ± SD	18.40 ± 5.04	18.67 ± 4.76	r = 0.010	0.463
HBI health practices, mean ± SD	17.70 ± 4.52	18.42 ± 4.08	r = 0.070	<0.001

Notes: HBI, Health Behaviour Inventory; SD, standard deviation; V, Cramér’s V; r, rank-based effect size. Percentages are based on valid responses within each profile. Comparisons of co-occurring behaviours were exploratory, and no global multiplicity correction was applied; raw *p*-values are therefore reported together with effect-size estimates.

## Data Availability

The data are not publicly available due to privacy and ethical restrictions related to research involving minors. Requests for access may be directed to the corresponding author and will be considered in accordance with the participants’ consent and applicable institutional requirements.

## Supplementary Materials

The following supporting information can be downloaded at: https://www.mdpi.com/article/10.3390/nu18172927/s1, Figure S1: Flow of participants from the source dataset to the final movement-behaviour profiles; Table S1: Operational definitions and analytical roles of variables included in the revised analysis; Table S2. Internal validation of candidate K-means solutions; Figure S2. Internal-validation metrics across candidate K-means solutions (k = 2–6); Table S3. Sensitivity of the retained two-profile solution to variable scaling; Table S4. Available-case denominators and missing observations for selected variables by movement-behaviour profile; Table S5. Full descriptive statistics for continuous outcomes according to movement-behaviour profile.
