# Supplementary material for: Physical Activity–Sedentary Behaviour Profiles and Co-Occurring Lifestyle Behaviours Among Adolescents: A Person-Oriented Analysis
Source: Nutrients. 2026 Sep 7;18(17):2927. doi: 10.3390/nu18172927 (PMC13567382; doi:10.3390/nu18172927)
Supplement: Supplementary file 1 [file nutrients-18-02927-s001.zip › nutrients-4497293-supplementary.pdf]

Supplementary Materials

Physical Activity-Sedentary Behaviour Profiles and Co-Occurring Lifestyle Behaviours among Adolescents: A Person-Oriented Analysis

Supplementary Methods

S1. IPAQ-LF Preprocessing and Analytical Sample

The movement-behaviour profiles were derived from four variables obtained from the International Physical Activity Questionnaire-Long Form (IPAQ-LF): weekly sedentary time, walking physical activity, moderate-intensity physical activity, and vigorous-intensity physical activity. These four variables were the only variables used to define the movement-behaviour profiles.

Before profile derivation, IPAQ-LF responses underwent the final data-quality screening and processing procedure applied in the revised analysis. For walking, moderate-intensity physical activity, and vigorous-intensity physical activity, reported durations exceeding 180 min/day were truncated to 180 min/day. Responses indicating no activity in a given component were coded as zero when this was consistent with the questionnaire response logic. When a valid weekly value could not be derived because frequency or duration information was missing or internally inconsistent, the corresponding value was treated as missing.

For each physical-activity component, reported minutes per day were multiplied by the reported number of days per week and summed across the relevant IPAQ-LF domains. Walking, moderate-intensity physical activity, and vigorous-intensity physical activity were expressed in minutes per week. Weekly sedentary time was calculated as five times the reported weekday sitting time plus two times the reported weekend-day sitting time and was likewise expressed in minutes per week.

No missing-value imputation was performed for any of the four profile-defining variables. A complete-case requirement was applied to profile derivation: participants were eligible for clustering only if complete and analytically valid weekly values were available for sedentary time, walking, moderate-intensity physical activity, and vigorous-intensity physical activity after final data processing.

The source project dataset comprised 3307 adolescents. Following IPAQ-LF processing, 1932 participants fulfilled the analytical eligibility criteria and constituted the primary clustering sample. The remaining 1375 participants (41.58% of the source dataset) were not included in profile derivation because at least one of the four required IPAQ-LF-derived variables was missing, incomplete, internally inconsistent, or otherwise did not meet the final analytical eligibility criteria.

S2. Cluster Derivation, Internal Validation, and Across-Initialisation Reproducibility

Empirical movement-behaviour profiles were derived using k-means clustering. Because the four profile-defining variables differed substantially in dispersion and k-means clustering is based on Euclidean distances, each variable was z-standardised before clustering. Standardisation placed sedentary time, walking, moderate-intensity physical activity, and vigorous-intensity physical activity on a comparable scale and reduced the possibility that cluster allocation would be disproportionately influenced by the variable with the greatest numerical dispersion.

Candidate solutions ranging from two to six clusters (k = 2-6) were evaluated. Initial cluster centres were selected randomly, and 100 initialisations were performed for each clustering procedure to reduce dependence on a particular starting configuration.

Internal validity was assessed using three complementary indices: the average silhouette coefficient, the Calinski-Harabasz index, and the Davies-Bouldin index. Higher silhouette and Calinski-Harabasz values indicate more favourable separation and compactness, whereas lower Davies-Bouldin values indicate a more favourable solution. Stability of cluster membership across repeated clustering solutions was assessed using the Adjusted Rand Index (ARI), with higher values indicating greater agreement between solutions.

The number of clusters was not selected on the basis of a single validation index. Instead, the retained solution was determined from the combined evidence regarding cluster separation, compactness, stability, parsimony, and substantive interpretability. On this basis, the two-cluster solution was retained for the primary analysis. Full internal-validation results for candidate solutions from k = 2 to k = 6 are provided in Supplementary Table S2.

A separate preprocessing sensitivity analysis compared the retained two-cluster solution based on z-standardised variables with a two-cluster solution derived from the original unstandardised minute-per-week

values. Agreement between these solutions was quantified using the ARI and is reported in Supplementary Table S3.

S3. Statistical Treatment of Co-Occurring Behaviours

Variables not used to derive the movement-behaviour profiles were analysed as co-occurring characteristics of the retained profiles. Categorical variables were summarised as numbers and percentages based on valid observations within each profile and were compared using Pearson's chi-square test. The phi coefficient (φ) was reported as the effect-size measure for 2 × 2 categorical comparisons, whereas Cramér's V was used for categorical tables with more than two categories.

Continuous variables were summarised using means and standard deviations and, where informative for non-normal distributions, medians and interquartile ranges. Between-profile comparisons of continuous outcomes were performed using the Mann-Whitney U test, with the rank-based effect size r reported to quantify the magnitude of the difference.

The four movement-behaviour variables used to derive the profiles were presented descriptively and were not subjected to inferential testing as independent evidence of between-profile differences. Because the retained solution comprised only two profiles, no post hoc pairwise procedures were required.

For sociodemographic, anthropometric, sleep, television/computer-use, internet-related, dietary, and Health Behaviour Inventory variables, available-case analysis was used. No missing-value imputation was performed; therefore, denominators could vary between outcomes. Profile-specific valid and missing observations for selected variables are reported in Supplementary Table S4.

Comparisons of co-occurring behaviours were considered exploratory. No global multiplicity correction was applied. Raw two-sided p-values were therefore reported together with effect-size estimates, and interpretation prioritised the magnitude of observed effects and consistency of the overall behavioural pattern rather than statistical significance alone. A conventional threshold of $p < 0.05$ was retained for statistical reporting but was not interpreted as confirmatory evidence in isolation.

Figure S1. Flow of participants from the source dataset to the final movement-behaviour profiles.

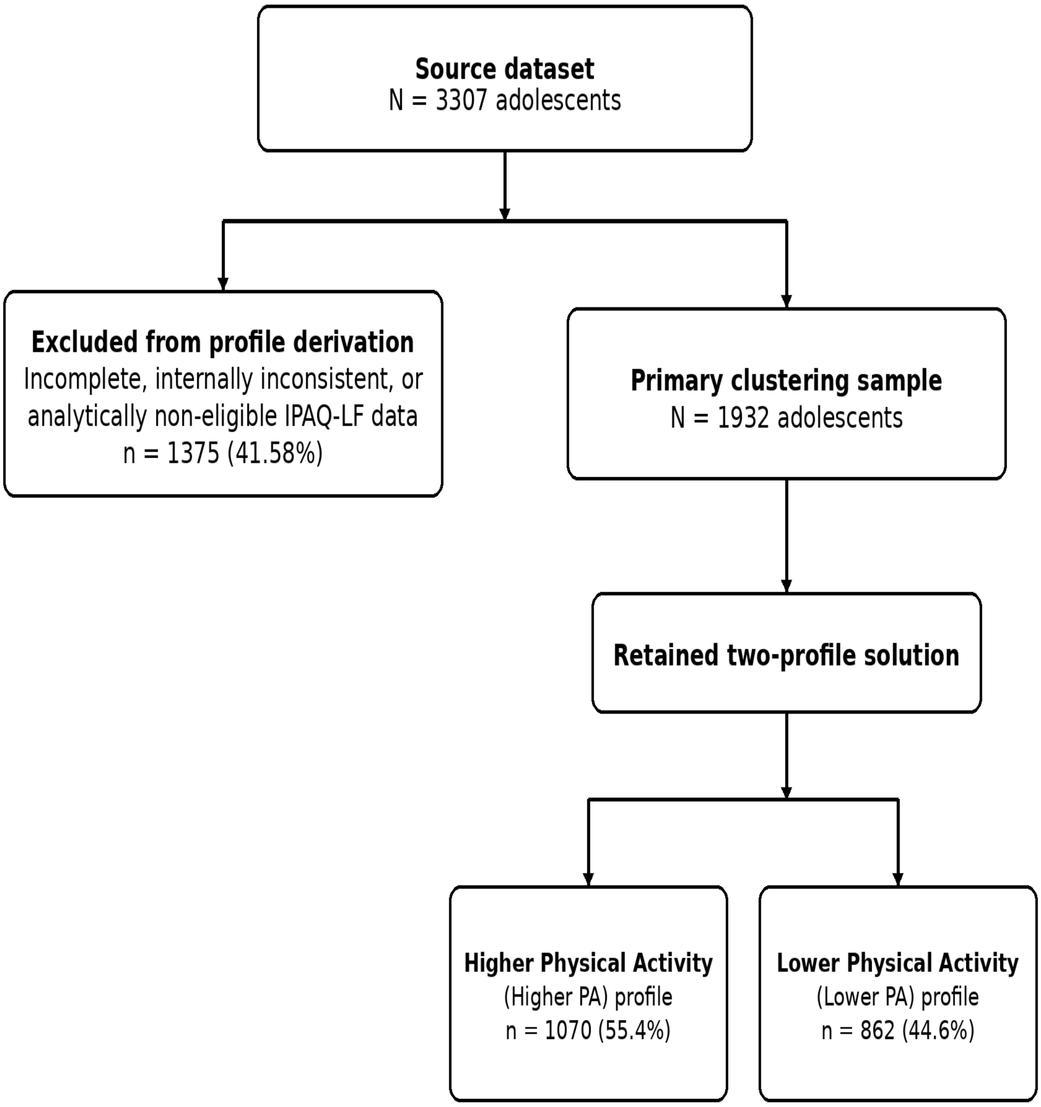

The source dataset comprised 3307 adolescents. After final preprocessing of the International Physical Activity Questionnaire-Long Form (IPAQ-LF), 1375 participants were excluded from profile derivation because at least one of the four required movement-behaviour variables (sedentary time, walking, moderate-intensity physical activity, or vigorous-intensity physical activity) was missing, incomplete, internally inconsistent, or otherwise analytically non-eligible. The final clustering sample therefore comprised 1932 adolescents. K-means clustering retained a two-profile solution, including 1070 adolescents in the Higher Physical Activity profile and 862 adolescents in the Lower Physical Activity profile. Percentages for excluded participants are based on the source dataset (N = 3307), whereas percentages for the retained profiles are based on the primary clustering sample (N = 1932).

Table S1. Operational definitions and analytical roles of variables included in the revised analysis.

Variable	Source	Operational definition	Analytical format	Analytical role	Missing-data strategy
Sedentary time	IPAQ-LF	Weekly sedentary time calculated as 5 × reported weekday sitting time + 2 × reported weekend-day sitting time after final data-quality screening.	Continuous, min/week	Profile-defining	Complete-case: valid value required for profile derivation; no imputation
Walking	IPAQ-LF	Reported min/day × days/week, summed across relevant IPAQ-LF domains. Durations >180 min/day were truncated to 180 min/day before calculation.	Continuous, min/week	Profile-defining	Complete-case: valid value required for profile derivation; no imputation
Moderate-intensity physical activity	IPAQ-LF	Reported min/day × days/week, summed across relevant IPAQ-LF domains. Durations >180 min/day were truncated to 180 min/day before calculation.	Continuous, min/week	Profile-defining	Complete-case: valid value required for profile derivation; no imputation
Vigorous-intensity physical activity	IPAQ-LF	Reported min/day × days/week, summed across relevant IPAQ-LF domains. Durations >180 min/day were truncated to 180 min/day before calculation.	Continuous, min/week	Profile-defining	Complete-case: valid value required for profile derivation; no imputation
Sex	Project questionnaire	Self-reported sex.	Categorical	Profile characterisation / sociodemographic comparison	Available-case; no imputation
Age	Project questionnaire	Self-reported age; study population restricted to second-year secondary-school students aged 15-17 years.	Continuous / descriptive	Sample characterisation; not included in clustering	Available-case; no imputation
BMI	Self-reported height and body mass	Body mass (kg) divided by height squared (m²).	Continuous, kg/m²	Profile characterisation / anthropometric comparison	Available-case; no imputation
School type	Project questionnaire	General secondary, technical secondary, or stage I vocational school.	Categorical	Profile characterisation / sociodemographic comparison	Available-case; no imputation
Place of residence	Project questionnaire	Rural area, small/medium-sized town, or large city.	Categorical	Profile characterisation / sociodemographic comparison	Available-case; no imputation
Father's education	Project questionnaire	Elementary/vocational, secondary, or university education.	Categorical	Profile characterisation / socioeconomic comparison	Available-case; no imputation
Mother's education	Project questionnaire	Elementary/vocational, secondary, or university education.	Categorical	Profile characterisation / socioeconomic comparison	Available-case; no imputation
School-day sleep duration	KomPAN	Average reported daily sleep duration on school days.	<7, 7-8, ≥9 h/day	Co-occurring comparison	Available-case; no imputation
Weekend sleep duration	KomPAN	Average reported daily sleep duration on days off/weekend days. The ≥9 h/day category was not prespecified as unfavourable.	<7, 7-8, ≥9 h/day	Co-occurring comparison	Available-case; no imputation
Television/computer use	KomPAN	Daily time spent watching television or using a computer; not interpreted as total contemporary screen exposure.	<2, ≥2 to <4, ≥4 to <6, ≥6 h/day	Co-occurring comparison	Available-case; no imputation
Internet-related risk	TPUI-22	Total TPUI-22 score categorised according to the study scoring system; very-low and low categories combined for analysis.	Very low/low, moderate, high, very high	Co-occurring comparison	Available-case; no imputation
Between-meal snacking	KomPAN	Reported frequency of eating between meals, collapsed into three analytical categories.	Never/1-3 times per month; once/several times per week; once/several times per day	Co-occurring comparison	Available-case; no imputation
Out-of-home meal consumption	KomPAN	Reported frequency of consuming meals outside the home, collapsed into three analytical categories.	Never/1-3 times per month; once/several times per week; once/several times per day	Co-occurring comparison	Available-case; no imputation

Variable	Source	Operational definition	Analytical format	Analytical role	Missing-data strategy
Energy-drink intake	KomPAN	Reported consumption frequency converted to daily equivalents using KomPAN conversion factors.	Continuous, times/day	Co-occurring comparison	Available-case; no imputation
Pro-Healthy Diet Index (pHDI-10)	KomPAN	Sum of consumption frequencies for 10 food groups represented in the pro-healthy index, converted to a 0-100 scale. Higher values indicate more frequent consumption of foods included in the index.	Continuous score, 0-100	Co-occurring comparison	Available-case; no imputation
Non-Healthy Diet Index (nHDI-14)	KomPAN	Sum of consumption frequencies for 14 food groups represented in the non-healthy index, converted to a 0-100 scale. Higher values indicate more frequent consumption of foods included in the index.	Continuous score, 0-100	Co-occurring comparison	Available-case; no imputation
HBI preventive behaviours	Health Behaviour Inventory	Six-item domain score; higher values indicate more frequent self-reported health-promoting behaviours.	Continuous, 6-30	Co-occurring comparison	Available-case; no imputation
HBI nutritional behaviours	Health Behaviour Inventory	Six-item domain score; higher values indicate more frequent self-reported health-promoting behaviours.	Continuous, 6-30	Co-occurring comparison	Available-case; no imputation
HBI positive mental attitude	Health Behaviour Inventory	Six-item domain score; higher values indicate more frequent self-reported health-promoting behaviours.	Continuous, 6-30	Co-occurring comparison	Available-case; no imputation
HBI health practices	Health Behaviour Inventory	Six-item domain score; higher values indicate more frequent self-reported health-promoting behaviours.	Continuous, 6-30	Co-occurring comparison	Available-case; no imputation

Notes: IPAQ-LF, International Physical Activity Questionnaire-Long Form; PA, physical activity; BMI, body mass index; TPUI-22, Problematic Internet Use Test; HBI, Health Behaviour Inventory; pHDI-10, Pro-Healthy Diet Index; nHDI-14, Non-Healthy Diet Index. The four IPAQ-LF-derived movement variables were the only variables used to derive the movement-behaviour profiles. Participants were included in clustering only when complete and analytically eligible values were available for all four profile-defining variables; no imputation was performed. All variables used solely for profile characterisation or co-occurring comparisons were analysed using available observations without imputation. The pHDI-10 and nHDI-14 were treated as separate dimensions of consumption frequency rather than opposite ends of a single dietary-quality continuum.

Table S2. Internal validation of candidate K-means solutions.

Number of clusters (k)	Silhouette coefficient ↑	Calinski-Harabasz index ↑	Davies-Bouldin index ↓	Across-initialisation ARI ↑
2	0.294	880.20	1.397	0.999
3	0.284	774.64	1.308	0.759
4	0.269	751.94	1.248	0.810
5	0.273	719.15	1.234	0.706
6	0.285	721.78	1.113	0.696

Notes: Higher values of the silhouette coefficient, Calinski-Harabasz index, and Adjusted Rand Index (ARI) indicate more favourable solutions, whereas lower values of the Davies-Bouldin index are preferred. No single validation criterion was treated as definitive. The two-cluster solution showed the highest silhouette coefficient, highest Calinski-Harabasz index, and highest stability, whereas the Davies-Bouldin index favoured more complex solutions. Considering cluster separation, compactness, stability, parsimony, and substantive interpretability jointly, the two-cluster solution was retained for the primary analysis. ARI quantifies agreement between clustering solutions obtained across repeated random initialisations using the same analytical sample. It reflects computational reproducibility with respect to initialisation and does not assess stability under sampling variability.

The retained k = 2 solution therefore represented the strongest overall compromise across internal-validation criteria and stability, despite only modest absolute cluster separation.

Figure S2. Internal-validation metrics across candidate K-means solutions (k = 2-6).

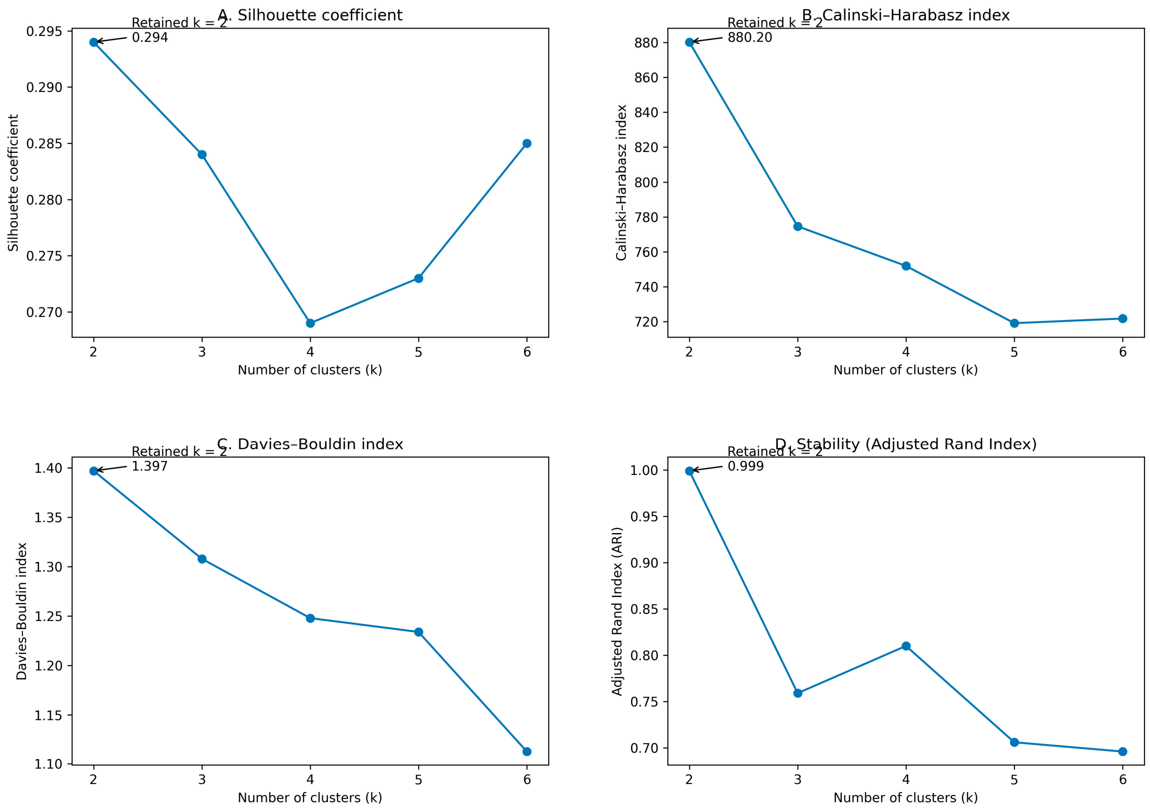

Panels show (A) silhouette coefficient, (B) Calinski-Harabasz index, (C) Davies-Bouldin index, and (D) Adjusted Rand Index (ARI) used to assess cluster stability. Higher silhouette, Calinski-Harabasz, and ARI values indicate more favourable solutions, whereas lower Davies-Bouldin values are preferred. The two-cluster solution showed the highest silhouette coefficient (0.294), Calinski-Harabasz index (880.20), and stability (ARI = 0.999), while the Davies-Bouldin index favoured more complex solutions. Considering separation, compactness, stability, parsimony, and interpretability jointly, k = 2 was retained for the primary analysis. Panel D presents the ARI used to assess reproducibility across repeated random initialisations within the same analytical sample.

Table S3. Sensitivity of the retained two-profile solution to variable scaling.

Analysis	Input metric	Observed clustering behaviour	Agreement with primary solution	Interpretation
Primary analysis	z-standardised variables	Sedentary time, walking, moderate-intensity physical activity, and vigorous-intensity physical activity contributed on a comparable scale to Euclidean distances.	Reference solution	Retained as the primary analysis because the four variables differed markedly in dispersion and K-means is distance-based.
Sensitivity analysis	Raw min/week values	Cluster allocation was predominantly influenced by sedentary time, which had greater numerical dispersion than the remaining movement-behaviour variables.	ARI = 0.008	Very low agreement with the primary solution, indicating substantial sensitivity of profile assignment to variable scaling.

Notes: ARI, Adjusted Rand Index. The sensitivity analysis repeated the retained two-cluster procedure using the original unstandardised minute-per-week values. An ARI of 0.008 indicates almost no agreement in individual profile assignment between the raw-data and z-standardised solutions. The sensitivity analysis was used to assess the dependence of the K-means solution on preprocessing and was not treated as an alternative primary classification. The z-standardised solution was retained because standardisation reduced disproportionate influence of variables with greater dispersion and allowed all four movement-behaviour dimensions to contribute on a comparable scale to the Euclidean-distance calculation.

The very low agreement between the two preprocessing approaches indicates that variable scaling materially influenced the empirical profile structure and should therefore be considered when interpreting the generalisability of the retained solution.

Table S4. Available-case denominators and missing observations for selected variables by movement-behaviour profile.

Variable	Higher PA valid / missing (%)	Lower PA valid / missing (%)	Total valid	Total missing	Absolute difference in missingness, percentage points
Sex	1070 / 0 (0.00%)	862 / 0 (0.00%)	1932	0	0.00
School type	1008 / 62 (5.79%)	821 / 41 (4.76%)	1829	103	1.03
Place of residence	856 / 214 (20.00%)	724 / 138 (16.01%)	1580	352	3.99
Father's education	787 / 283 (26.45%)	658 / 204 (23.67%)	1445	487	2.78
Mother's education	816 / 254 (23.74%)	675 / 187 (21.69%)	1491	441	2.05
School-day sleep duration	1016 / 54 (5.05%)	822 / 40 (4.64%)	1838	94	0.41
Weekend sleep duration	1012 / 58 (5.42%)	822 / 40 (4.64%)	1834	98	0.78
Television/computer use	1026 / 44 (4.11%)	825 / 37 (4.29%)	1851	81	0.18
Internet-related risk	1070 / 0 (0.00%)	862 / 0 (0.00%)	1932	0	0.00
Between-meal snacking	1046 / 24 (2.24%)	841 / 21 (2.44%)	1887	45	0.20
Out-of-home meal consumption	1070 / 0 (0.00%)	862 / 0 (0.00%)	1932	0	0.00
Pro-Healthy Diet Index	1064 / 6 (0.56%)	853 / 9 (1.04%)	1917	15	0.48
Non-Healthy Diet Index	1064 / 6 (0.56%)	853 / 9 (1.04%)	1917	15	0.48

Notes: PA, physical activity. Percentages of missing observations were calculated within the final profile sizes (Higher PA, n = 1070; Lower PA, n = 862). The final column presents the absolute difference in missing-data percentage between profiles and is descriptive only; no inferential test of differential missingness was performed. Variables for which final profile-specific valid-N information was not available were not included. All between-profile analyses of these variables used available observations without imputation.

Table S5. Full descriptive statistics for continuous outcomes according to movement-behaviour profile.

Variable	Higher PA mean ± SD	Higher PA median (IQR)	Lower PA mean ± SD	Lower PA median (IQR)	Effect size r	p-value
BMI, kg/m²	21.82 ± 4.36	21.13 (4.24)	21.16 ± 3.47	20.76 (4.11)	0.080	0.001
Energy-drink intake, times/day	0.25 ± 0.49	0.06 (0.14)	0.18 ± 0.40	0.06 (0.14)	0.080	0.001
Pro-Healthy Diet Index (pHDI-10)	21.15 ± 11.74	18.70 (15.05)	18.71 ± 10.16	16.90 (14.00)	0.100	<0.001
Non-Healthy Diet Index (nHDI-14)	19.41 ± 11.59	16.71 (12.54)	17.32 ± 9.39	15.71 (10.79)	0.090	<0.001
HBI preventive behaviours	17.07 ± 5.04	17.00 (7.00)	17.43 ± 4.47	18.00 (6.00)	0.040	0.122
HBI nutritional behaviours	17.70 ± 4.83	18.00 (6.00)	17.37 ± 4.47	17.00 (6.00)	0.040	0.058
HBI positive mental attitude	18.40 ± 5.04	19.00 (7.00)	18.67 ± 4.76	19.00 (7.00)	0.010	0.463
HBI health practices	17.70 ± 4.52	18.00 (6.00)	18.42 ± 4.08	18.00 (5.00)	0.070	<0.001

Notes: PA, physical activity; BMI, body mass index; SD, standard deviation; IQR, interquartile range; HBI, Health Behaviour Inventory; pHDI-10, Pro-Healthy Diet Index; nHDI-14, Non-Healthy Diet Index; r, rank-based effect size for the Mann-Whitney U comparison. P-values are two-sided, unadjusted, and should be interpreted as exploratory. No global multiplicity correction was applied. Effect-size magnitude and consistency of the overall pattern should therefore be prioritised over statistical significance alone.

Although several comparisons reached conventional statistical significance, all observed effect sizes were small ($r \leq 0.100$).
